# Why should we screen for arterial hypertension in children and adolescents?

**DOI:** 10.1007/s00467-017-3739-8

**Published:** 2017-07-17

**Authors:** Mieczysław Litwin

**Affiliations:** Department of Nephrology and Arterial Hypertension, The Children’s Memorial Health Institute, 04-730 Aleja Dzieci Polskich, 20 Warsaw, Poland

## Introduction

Screening is a strategy used in a given population to identify an unrecognized disease in otherwise healthy people without any signs or symptoms. This can include individuals with pre-symptomatic or unrecognized symptomatic disease. As such, screening tests are somewhat unique in that they are performed on persons apparently in good health. The aim of screening is to diagnose disease at its earliest, preclinical stage and, if possible, to initiate treatment or lifestyle intervention before irreversible complications develop. However, screening may lead to overdiagnosis, and may occasionally result in adverse effects caused by unnecessary diagnostic tests, false diagnosis and even unnecessary treatment. On the other hand, screening tests with low sensitivity may create a false sense of security and, therefore, they must be characterized by both good sensitivity and specificity. In 1968 the World Health Organization published guidelines on the “Principles and practice of screening for disease”. Although these principles were modified in 2008 and adjusted to screening based on genomic technologies, the main principles of screening, known as Wilson’s criteria, are still valid (Table 1) [1].

**Table 1:** World Health Organization screening criteria

World Health Organization screening criteria^a^
1. The condition should be an important health problem.
2. There should be a treatment for the condition.
3. Facilities for diagnosis and treatment should be available.
4. There should be a latent stage of the disease.
5. There should be a test or examination for the condition.
6. The test should be acceptable to the population.
7. The natural history of the disease should be adequately understood.
8. There should be an agreed policy on whom to treat.
9. The total cost of finding a case should be economically balanced in relation to medical expenditure as a whole.
10. Case-finding should be a continuous process, not just a “once and for all” project

^a^Adapted from Wilson and Jungner [1] http://apps.who.int/iris/bitstream/10665/37650/17/WHO_PHP_34.pdf, used with permission

Screening tests are one of the main tasks of paediatric care but are performed even before birth, during the prenatal period in pregnant women. Screening in general and particularly in paediatrics should be done during routine, regular well-child visits [2]. The goal of a well-child visit is to detect disease and to prevent disease, which means to detect it at its very early, preclinical stage, to promote a healthy lifestyle and to effect anticipatory guidance.

After more than 100 years of hypertension research, it is well known that elevated blood pressure (BP) caused by primary hypertension (PH) is the most important potentially reversible cardiovascular risk factor in the general population [3]. Thus, when the aim is to detect elevated BP, to prevent cardiovascular disease (CVD) and to promote a healthy lifestyle, the latter being equivalent to non-pharmacological treatment of arterial hypertension (AH), the BP must be measured first. However, there are a number of basic questions which should also be answered. First, is elevated BP in the paediatric population sufficiently prevalent to justify population screening? Second, is elevated BP in children and in adolescents a risk factor for CVD in adulthood? Third, is the treatment of AH in children and in the general population an effective strategy to reduce cardiovascular death and cardiovascular events? Fourth, is BP screening in children safe? In the following sections I attempt to answer these four questions and comment upon whether BP screening should be performed in children and adolescents.

## Is elevated BP in the paediatric population sufficiently prevalent to justify population screening? Prevalence, incidence and aetiology of AH in children and adolescents

### Prevalence of AH in the paediatric population

Results of large epidemiological studies indicate that 30–40% of the adult population above 20 years of age suffer from AH [4]. However, there is a general trend towards an increasing prevalence of AH with age. In younger adults, aged between 18 and 40 years, the prevalence of AH is similar to that in adolescents, i.e. about 10–15%, increasing linearly with age [5, 6]. AH is associated with significant mortality and morbidity caused by stroke, heart failure and ischaemic heart disease. In some parts of the world, AH may cause more than 20 years of disability [7].

It is estimated that the prevalence of AH among children and adolescents aged between 0 and 18 years is 3–5% [8]. However, the prevalence of AH is very low in neonates and infants (ranging from 0.2% in otherwise healthy newborns to 0.8% in premature infants hospitalized in neonatal intensive care units) and increases with age, reaching about 10–11% in 18-year-old adolescents (diagnosis based on elevated BP during three independent visits) [9–12]. A recent analysis of worldwide BP trends during the last four decades, including adolescents up to the age of 18 years, showed that average BP values have significantly decreased in high-income countries, increased in low-income countries and have remained persistently high in central and eastern Europe [13]. The largest decrease of systolic BP (SBP) was observed in high-income Asia–Pacific countries, being 3.2 mmHg per decade for women and 2.4 mmHg per decade for men. In contrast, in low-income countries (southeast Asia, sub-Saharan Africa, Oceania) both mean SBP and diastolic BP have increased. In general, in 2015 in a number of high-income countries (Canada, South Korea, UK, USA, Singapore, Australia) age-standardized prevalence of AH was <13% in women and <19% in men. In contrast, in central and eastern Europe (Croatia, Latvia, Lithuania, Slovenia and Hungary) the prevalence was >35% in men. These findings underline the effect of socioeconomic status on BP. Similar ethnicity- and socioeconomic status-dependent differences were noted among children and adolescents in the USA, where the highest prevalence of AH and high-normal BP were observed among African-Americans (15.3%) and Mexican-Americans (11.5%) and the lowest were observed among non-Hispanic whites (9.4%) and Asians (8.5%) [14]. Similar results indicating a relationship between low socioeconomic status and AH were found among children and adolescents in Poland [15] where the prevalence of AH (BP measured twice on 3 independent occasions) ranged from 5.6% in 10-year-old children to 7.9% in 18-year-old adolescents. However, the prevalence of AH was higher in rural areas (9.9%) than in large cities (4.4%) and among children whose parents were not well educated (7.2 vs. 3.5% in children of well-educated parents), and it was associated with lower income (5.3 vs. 3.4%) [15].

A recently published report from the USA shows that elevated BP, defined as an average value obtained from three measurements done on one occasion and higher than the 90th percentile, was found in >10% of children aged between 8 and 17 years [14]. In a study from Poland, about 6% of all subjects from the general paediatric population aged between 3 and 18 years had BP above the 95th percentile when it was measured three times on one occasion, with the average of the second and third measurements used for analysis [16]. The exact prevalence of AH diagnosed as per the binding definition is difficult to estimate because most data are based on measurements carried out during one visit. In a study of 14.5-year-old adolescents from the general population in which AH was diagnosed when the mean value of three independent BP measurements was ≥95th percentile, prehypertension was diagnosed in 9% of boys and 7.7% of girls, and AH was diagnosed in 12.3% of all cases (11.4% in boys and 13.1% in girls) [17]. In the same study, stage 1 AH was found in 8.6% of boys and 9.7% of girls and stage 2 AH in 2.9 and 3.4% of boys and girls, respectively [17].

In the older group of adolescents, diagnosis of AH may depend on the criteria applied. In 2016 the European Society of Hypertension modified the definition and classification of AH in adolescents aged ≥16 years, proposing instead that in this group of adolescents, both girls and boys, adult criteria for the diagnosis and classification of AH should be used irrespective of percentile values of BP [18]. This modification has important practical consequences. Thus, diagnosis of AH among 18-year-old adolescents may differ in relation to the cut-off criteria used. In one study conducted in Poland, the prevalence of AH among 18-year-old students was 14.7% (21.9% among boys and 6.6% among girls) when paediatric criteria, based on the 95th percentile of BP values, were used; when adult criteria for the diagnosis of AH were applied, AH was diagnosed in 9% (19.5% in boys and 0.9% in girls) [12].

### Incidence of AH in children and adolescents

Data are scarce on the incidence of AH in childhood. Most studies have focused on the evolution from prehypertension to sustained AH among adolescents and the incidence of AH in children from groups with higher risk factor levels for CVD, such as prematurity, type 1 and type 2 diabetes mellitus (DM), and status post repair of aortic coarctation (CoA). It is estimated that among adolescents from the general population the rate of progression from normotension to AH, as confirmed by three measurements on three independent visits, is 0.3% per year, while the incidence rate among adolescents who were prehypertensive is 1.1% per year [19]. The greatest risk of development of AH was noted among adolescents in whom BP values were in the prehypertensive or hypertensive range before screening, with an incidence rate of 1.4% per year, the same as among adults with optimum BP [20]. An unexpectedly high incidence rate was found in the IDEFICS study [21]. However, because in the IDEFICS study BP was measured two times on one occasion, AH could not be diagnosed based on the binding definition. Thus, only development of “pre-high blood pressure” (preHBP), i.e. BP above the 90th percentile and below 95th percentile, and so-called “high blood pressure” (HBP), i.e. BP in the hypertensive range (>95th percentile) could be estimated [21]. It was found that the incidence of preHBP and HBP within the hypertensive range per year was 121/1000 children and 110/1000 children, respectively. Even if incidence rates found in the IDEFICS study are overestimated, the important finding is that sedentary behaviour and low physical activity were associated with significantly increased incidence of preHBP and HBP.

In a prospective study of adolescents with type 2 DM, 11.6% were hypertensive at baseline and 33.8% after 3.9 years, which gives a 4% per year incidence rate of AH [22]. Elevated BP is also one of the main findings in children with CoA. After successful repair, residual hypertension is still present in about 25% of children. However, it has been shown that 16.7% of children will develop late AH after 13 years of age, which gives an incidence rate of about 1.3% per year [23]. AH is a common complication of chronic kidney disease (CKD). A recently published analysis of data from 545 children with CKD stage 2–5 revealed that 26.1% of children had uncontrolled hypertension (confirmed by 24-h ambulatory BP monitoring) and that the prevalence increased from 24.4% in stage 3 to 47.4% in stage 5 CKD [24].

### Aetiology of AH in children and adolescents

Although historically the most prevalent form of AH in childhood was secondary AH, this has changed in the last two decades. PH is now starting to become the dominant cause of AH in children older than 6 years of age, and its prevalence is at least the same as that of secondary hypertension. However, in one study the prevalence of secondary AH among teenagers referred to an ambulatory hypertension clinic was 51% [25]. Importantly, in this study children referred to the outpatient hypertension clinic were diagnosed as hypertensive during routine screening and not because of symptomatic AH. In all groups with secondary AH, renal aetiology (including renovascular disease) dominated (34%), followed by respiratory causes (20%), medications (13%) and neurological disease (12%). However, in infants and children up to 5 years of age, respiratory diseases dominated (60% in the first year of life and 36% in children aged 1–5 years), followed by renal causes of AH (13% in the first year of life and 34% in children aged 1–5 years) [25]. The recent rise in the prevalence of PH in childhood and adolescence is strictly associated with the obesity epidemic, while the dominant intermediate phenotype of a hypertensive adolescent is altered body composition with visceral obesity and metabolic abnormalities typical of metabolic syndrome. The general rule is that the younger the child and the more severe the AH, the greater the probability of secondary AH—which is why hypertensive prepubertal children and children with stage 2 AH irrespective of age need deeper diagnostic investigations [18, 26].

To summarize the above, the prevalence of AH increases with age and already poses a significant problem among school-age children. In adolescents, the prevalence of AH is similar to that in young adults. The incidence rate of AH in the general paediatric population is lower than that in adults, but among adolescents who have BP in the high-normal/prehypertensive range it is similar to the incidence of AH observed in adulthood. Among children from risk groups, such as diabetic children, children with CKD and children post CoA repair, both the prevalence and incidence of AH is much greater than that in the general paediatric population. Although PH is rare in preschool children its prevalence increases with age, and it is now the dominant form of AH among adolescents, and in the general paediatric population its prevalence is similar to that of secondary forms of AH. However, secondary AH is often still the cause of AH among asymptomatic teenagers referred to hypertension clinics due to elevated BP found during routine screening.

## Is elevated BP in children and adolescents a risk factor for CVD in adulthood?

Primary hypertension, the main form of AH in adolescents and adults, is a slowly progressing disease which usually does not lead to clinically overt CVD in childhood and adolescence. The exception is secondary AH. Early diagnosis of AH may prevent the development of the most severe complications of AH. There is no controversy regarding the treatment and screening for secondary AH in children from risk groups, such as children with CKD or children having had CoA repair. It is well known that secondary AH is more severe, is associated with target organ damage (TOD) and leads to serious complications already in childhood if left untreated. Early treatment might not only protect against TOD but also delay progression of CKD [27, 28]. However, it is the diagnosis of PH and its treatment that remains a matter of controversy. Should we really check for elevated BP in otherwise healthy children and should we diagnose PH at its earliest stage?

## Is PH in childhood a benign disease?

Primary hypertension is not an isolated, haemodynamic phenomenon. It is a complex neuro-immunological and metabolic disease in which BP is linked with other cardiovascular risk factors. PH in childhood presents itself with a typical intermediate phenotype. It is usually diagnosed after 6 years of age, and in most cases at a prepubertal or pubertal stage, and is strongly associated with disturbed body composition, with visceral obesity and metabolic abnormalities typical of metabolic syndrome, while a tendency for elevated uric acid levels is regarded as the most typical biochemical abnormality [29–36]. Metabolic syndrome is present in about 15–20% of hypertensive children already at diagnosis [37, 38]. When more sophisticated diagnostic methods are used, accelerated biological maturation, significant alterations in the immune system and disturbed activity of the sympathetic nervous system can be observed [39–45]. These abnormalities indicate that treatment of PH and early CVD does not simply involve decreasing the BP, but rather decreasing exposure to interconnected cardiovascular neuro-immuno-metabolic risk factors. Data from a recently published NHANES study indicate that slightly more than one in ten children aged between 8 and 17 years had elevated BP [14]. However, what is additionally interesting is that one in five children manifested significant metabolic abnormalities. These results provide yet another piece of evidence that cardiovascular risk factors cluster together.

A roughly 10% prevalence of elevated BP in adolescents indicates that it is an important health problem. From a clinical point of view it is important to know whether elevated BP and PH in childhood is associated with TOD. There are a few markers of cardiovascular injury caused by AH, including left ventricular hypertrophy (LVH), subclinical arterial damage, expressed as increased carotid intima-media thickness (cIMT), increased stiffness of great arteries, expressed as carotid-femoral pulse wave velocity (PWV), and hypertensive injury of glomerular capillaries leading to albuminuria. Disturbed function of the endothelium, measured as flow-mediated dilation (FMD), is also regarded as secondary to hypertensive injury.

Although cardiac events are rare or have not been reported as yet in children with PH, TOD in the form of LVH and hypertensive arteriopathy are already observed in about 40% of children with PH at diagnosis [34, 46]. Importantly, severe LVH, i.e. left ventricular mass index (LVMi) exceeding the 99th percentile and greater than 51 g/m of height^2.7^, has been observed in 13–15% of children with PH already at diagnosis [46, 47]. A recently published meta-analysis showed that the LVMi in children with PH was on average greater by almost 7 g/m of height^2.7^ than that in normotensive children [48]. Interestingly, children with white coat hypertension also had significantly greater LVMi than normotensive children [48]. Similarly, average values of cIMT were found to be significantly greater in hypertensive children and adolescents, and about 40% of them had cIMT values diverging by more than 2 standard deviation scores from the median of normal. Moreover, hypertensive children manifested decreased arterial elasticity already at diagnosis of hypertensive disease and had faster PWV in comparison with normotensive children and children with white coat hypertension [48].

Adaptive changes in the arterial tree, described as increased cIMT, faster PWV and lower FMD, are typical characteristics of aged arteries and are the hallmarks of early vascular ageing (EVA). Thus, a child with PH may match all criteria of EVA [49]. The concept of EVA was introduced a few years ago, and it describes the main disturbances leading to elevated BP as observed with ageing. Increased cIMT, stiffer arteries and lower generation of nitric oxide, estimated as lower FMD, perpetuate a vicious circle and lead to a further increase of BP [50, 51]. Both arterial changes and neuro-immuno-metabolic abnormalities observed in children with PH indicate that hypertensive children manifest the early stage of CVD according to the EVA hypothesis. Thus, the next question is whether elevated BP in childhood, with its known consequences for the cardiovascular system observed already in childhood and adolescence, results in a risk for CVD in adulthood.

## Elevated BP in childhood and adolescence and cardiovascular risk in adulthood

Prospective population studies provide data on the significance and consequences of exposure to different risk factors. Data from such studies, analysing the effects of exposure to cardiovascular risk factors, such as elevated BP in childhood on cardiovascular risk and cardiovascular complications, have been available for a few decades. One of the analyses from the Bogalusa Heart Study involving 824 adults at the mean age of 36 years revealed that elevated BP in childhood predicted concentric LVH [52]. In a more detailed analysis of data from the Bogulasa Heart Study, a relationship between long-lasting exposure to elevated BP and other cardiovascular risk factors and LVH in adulthood was calculated [53]. The first examinations were made in children at a mean age of 9.8 years and the last examination was done at a mean age of 37 years. It transpired that both adiposity and elevated BP in childhood and long-lasting exposure to excessive adiposity and elevated BP were significantly associated with LVH in adulthood. Importantly, adiposity exerted a greater effect on LVH than did elevated BP. Recent data from the Cardia Study provides evidence that exposure to elevated BP starting from adolescence and young adulthood is associated not only with LVH but also with diastolic dysfunction of the left ventricle, as assessed by speckle tracking echocardiography in middle age (43–55 years) [54].

Associations between elevated BP in childhood and the development of coronary atherosclerosis were analysed in the Cardiovascular Risk in Young Finns Study. The results of this study showed that exposure to elevated BP in adolescence (12–18 years of age) independently predicted the presence of coronary artery calcifications 27 years later [55]. The same was observed in relation to subclinical hypertensive arterial injury expressed as increased cIMT. The effects of elevated BP starting from childhood on cIMT in adulthood were analysed by the International Childhood Cardiovascular Cohort Consortium (ICCCC). This cohort consisted of 4210 participants from four prospective studies (Bogalusa Heart Study, Muscatine Study, Cardiovascular Risk in Young Finns Study and Childhood Determinants of Adult Health study). The age of the participants at first examination was 9–18 years and then 23–46 years in adulthood [56, 57]. Subjects with persistently elevated BP from childhood to adulthood had the greatest values of cIMT, while those who were normotensive in childhood but had elevated BP in adulthood had lower cIMT than those persistently hypertensive from childhood but greater cIMT than those who had elevated BP in childhood but normal BP in adulthood. Those who had persistently normal BP had the lowest cIMT. Elevated BP in the ICCCC was defined as BP above the 90th percentile in childhood and above 120/80 mmHg in adulthood. However, when more strict criteria of elevated BP were used (above 95th percentile for children and above 140/90 mmHg for adults), the risk ratio for increased cIMT in subjects with persistently elevated BP or who became hypertensive as adults became greater. It must be emphasized here that the risk of development of carotid hypertensive arteriosclerosis was significantly reduced if elevated BP in childhood normalized in subsequent years.

To summarize, there is sufficient evidence that elevated BP and PH in childhood cause significant cardiovascular risk and promote the development of cardiovascular injury already in young and middle adulthood. Moreover, normalization of elevated BP in childhood decreases the risk of premature arteriosclerosis. Thus, both factors support the argument for both the early detection and treatment of childhood hypertension.

## Is treatment of AH in children effective in reducing the risk of cardiovascular death?

A lot of data shows that antihypertensive treatment in adults is not only effective in terms of decreasing BP but that it also leads to a decrease in complications caused by AH. A recent analysis of global data showed that in addition to an increase in life expectancy observed in the last two decades, there has also been a significant decrease in death rates caused by complications of hypertensive disease, such as stroke (both haemorrhagic and ischaemic) and hypertensive heart disease. These results suggest that both preventive and therapeutic measures in adults are effective in reducing the rates of CVD associated with AH, and mostly with PH [58]. Such measures are also cost-effective [59].

To date no prospective controlled studies have been reported showing that the treatment of AH in children reduces cardiovascular death. However, there are data from paediatric prospective, controlled studies showing the efficacy of antihypertensive treatment in some selected groups of patients. The most spectacular effects were documented in children with CKD, with controlled lowering of BP during therapy based on angiotensin-converting-enzyme inhibitors (ACEi) resulting in significant slowing of progression towards end-stage renal disease [28]. There are also data indicating that even relatively short treatment caused regression of LVH in a patient group, including children with secondary AH and PH [60, 61]. Analysis of data from the prospective observational Chronic Kidney Disease in Children (CKiD) study revealed that lowering the BP of children with CKD caused a decrease of LVMi [62]. In the controlled and prospective ESCAPE study, achievement of lower BP during treatment based on ramipril therapy resulted in improved myocardial function (midwall fractional shortening) [27]. Small controlled studies have shown that antihypertensive therapy in children with Williams syndrome and with Marfan syndrome slows the rate of enlargement of aortic diameter [63, 64].

Thus, there are no doubts regarding the need to measure BP in children from risk groups and/or with disorders directly associated with AH and those suspected of secondary AH. The main controversies revolve around children with PH in whom the severity of AH is lower and who, despite a quite high prevalence of TOD at presentation, do not manifest clinically significant complications of hypertensive disease until adulthood. The main counter-argument against BP screening to diagnose PH in childhood is the lack of evidence that early treatment will lead to a decrease of cardiovascular risk and will lower cardiovascular mortality [65]. However, as discussed above, there is a direct link between exposure to higher BP over a wide range from prehypertensive to hypertensive values starting from childhood and the development of significant TOD and CVD in early adulthood. These alterations in the cardiovascular system, expressed as LVH, deteriorated diastolic function of the left ventricle, hypertensive arteriopathy and coronary atherosclerosis, are established risk factors of cardiovascular morbidity and mortality. Moreover, data from observational studies indicate that antihypertensive treatment (both non-pharmacological and pharmacological) in children with PH is effective in terms of decreased BP, regression of TOD and normalization of metabolic abnormalities.

In a prospective study in hypertensive children, Assadi observed that 1 year of antihypertensive treatment based on ACEi and hydrochlorothiazide caused significant decreases of BP, LVMi, prevalence of LVH and microalbuminuria [66]. Kupferman et al. conducted a small study and found that antihypertensive treatment caused a significant decrease of LVMi [67]. In a series of reports from a prospective, observational study involving a group of 86 children with PH, we found that 1 year of antihypertensive treatment based on non-pharmacological therapy and ACEi or angiotensin receptor blocker caused a significant decrease of the BP and an almost complete disappearance of the severe ambulatory hypertension category (41.9 vs. 1.2%) [68]. Moreover, we also observed that the LVMi and the prevalence of LVH and severe LVH significantly decreased (11.6 vs. 1.2%) and the cIMT decreased [38]. Analysis of changes in left ventricular geometry in children with PH revealed normalization after 1 year of treatment [68].

Antihypertensive therapy is not only beneficial to the cardiac system of children with PH (both non-pharmacological and pharmacological), it is also associated with significant improvements in metabolic and immunological abnormalities. Some studies have reported a >50% decrease in the prevalence of metabolic syndrome (15 vs. 7%), normalization of highly sensitive C reactive protein and decreased levels of markers of oxidative stress, such as serum concentrations of oxidized low-density lipoprotein-cholesterol and thiobarbituric acid reacting substances (formed as a byproduct of lipid peroxidation) [38, 69]. In addition, after 1 year of treatment serum concentrations of asymmetric dimethylarginine, an endogenous inhibitor of nitric oxide synthesis, significantly decreased. A more sophisticated analysis of a small group of adolescents with PH treated only non-pharmacologically showed a significant decrease of BP accompanied by significant changes in the expression of the renin–angiotensin system genes in peripheral blood leucocytes [70].

In summary, these findings from paediatric prospective studies indicate that antihypertensive treatment is associated with beneficial effects both in terms of decreasing BP and leading to regression of hypertensive TOD. If obtained in young adults one decade older, the same results would be interpreted as excellent. However, it must be emphasized here that the possibility of achieving regression of TOD is greater when treatment is started in the early phase of disease.

## Is BP screening in children and adolescents safe?

To the best of this author’s knowledge, there are no reports suggesting that BP measurement during screening causes any harm—although at the same time it may lead to potential overdiagnosis of AH in a healthy child. There are two groups of children and adolescents in whom AH may be falsely diagnosed, namely neonates and small children and adolescents aged ≥16 years.

The number of unreliable BP readings is greatest among the youngest children, primarily due to false positive results of BP measurements most often caused by the white coat effect or arm movement during the BP measurement. The white coat effect caused by emotional stress is observed in all age groups, but is especially evident in neonates and small children. Blake et al. reported that in children in the first year of life, up to 41% of BP measurements were unreliable due to anxiety or movement during measurements [71]. Similarly, Nwankwo et al. documented that there is significant white coat effect present in neonates and that routine BP measurements gave significantly greater results than values obtained when a special protocol of BP measurement was used [72]. The white coat effect or lack of compliance during BP measurement was also observed even when BP was measured by specially trained staff. In our population study of over 8000 preschool children, BP measurements were unreliable in 20% of children in the third year of life; in all groups of children aged 3–6 years BP values were falsely increased in 9% [73]. Another potential risk factor for false diagnosis of AH is the inappropriate use and interpretation of data obtained from 24-h ambulatory blood pressure monitoring. False positive diagnosis of AH may lead to unnecessary diagnostic procedures and even treatments, which is why both the Fourth Task Report guidelines and guidelines of the European Society of Hypertension do not recommend universal screening of BP in children younger than 36 months [18, 26].

Another issue is the diagnosis of AH in adolescents. As mentioned previously, the diagnosis of AH in adolescents aged ≥16 years should be based on BP cut-off values, as in adults, i.e. ≥140/90 mmHg [18]. This problem is important, especially in girls aged 16–18 years in whom the difference between the adult cut-off for SBP (140 mmHg) and the 95th percentile may even reach 9 mmHg. The use of the paediatric definition may lead to diagnosis of AH in a 17-year-old girl with SBP values in the range of 132–135 mmHg.

To summarize BP measurement is a safe procedure. However, improper BP measurements and the wrong interpretation of obtained results may lead to an overdiagnosis of AH. Thus, it is important to decide in whom and how BP should be measured.

## Do not ask why we should screen for elevated BP in children. Ask only when we should start

Evidence-based decision-making upon reasonable BP screening in children should first answer the questions stated at the beginning of this review. The first question was whether the prevalence of AH in childhood and adolescence is sufficiently high to advocate population screening. The answer is that the prevalence of AH in childhood increases with age and is the same in 18-year-old adolescents as in young adults. Thus, AH in childhood and adolescence is an important problem from the public health point of view.

The second question was whether AH in childhood is a risk factor for CVD and cardiovascular complications. There is no doubt that secondary AH causes life-threatening complications, TOD and accelerated progression of CKD. There is also a large body of data indicating that PH causes significant TOD already at diagnosis in childhood or adolescence and increases cardiovascular risk in adulthood. Thus, the answer to this question is yes.

The third question was whether the treatment of AH in children is safe and effective in terms of decreasing cardiovascular risk. Data from both clinical drug trials and retrospective and prospective observational and interventional studies indicate that antihypertensive treatment is both safe and effective. Treatment of both secondary AH and PH results in a decrease of BP and regression of TOD. Moreover, in the case of PH, treatment causes the regression of both cardiovascular complications and immune-metabolic abnormalities and reduces total cardiovascular risk. Although studies conducted to date on the effects of antihypertensive treatment on the regression of TOD and metabolic abnormalities are relatively short and lasted 6–12 months, the results are unequivocal and document beneficial effects. Importantly, these beneficial effects were also observed with non-pharmacological treatment.

The fourth question was whether BP screening in children is safe. As described in the preceding section, BP measurements may give false positive results when taken and they may be interpreted inappropriately, thus possibly leading to unnecessary diagnostic procedures and treatment. Therefore, the answer is that BP screening is safe but may lead to overdiagnosis of AH when it is done in an inappropriate way and in an inappropriately chosen group of children, and when the data are not interpreted properly.

Therefore, to state the question properly, it is not ‘why should we screen for elevated BP in children?’, but rather ‘when should we start BP screening?’ It would appear that the guidelines of the Fourth Report and the paediatric guidelines of the European Society of Hypertension are both safe solutions, recommending population screening in children aged >3 years [18, 26]. However, in children with risk factors BP measurements should be performed routinely from birth.

According to the US Preventive Services Task Force (USPSTF) statement there is no evidence that routine screening of asymptomatic children and adolescents for PH and early diagnosis of PH will deliver any benefit [65]. However, here I have presented proof that PH in children and adolescents is the first stage of CVD, that it is already associated with significant hypertensive TOD at diagnosis and that early treatment leads to regression of TOD, reduction of cardiovascular risk factors and decrease of cardiovascular risk in adulthood. Thus, it is clear that early diagnosis and treatment are beneficial strategies. The other drawback of the USPSTF statement is that the discussion concerns only PH. However, the aim of BP screening in asymptomatic children is not only to detect children with PH but also to detect asymptomatic children with secondary AH before it becomes symptomatic. As already mentioned, despite PH being the dominant form of AH in adolescents and adults, most of the children referred to hypertension clinics have secondary AH. Thus, screening to diagnose elevated BP will also lead to the diagnosis of asymptomatic children and adolescents with secondary AH. Not only personal experience but also published data on the aetiology of AH in children and adolescents clearly show that secondary AH may not be symptomatic and that it is diagnosed during routine BP screening in asymptomatic, otherwise healthy children [25]. Furthermore, BP screening is the best strategy to diagnose not only PH but also secondary AH. Thus, abandoning BP screening in asymptomatic children and adolescents will lead not only to delayed diagnosis and treatment of PH but also to secondary AH.

## Conclusions

A recently published statement from the USPSTF brings into question the legitimacy of BP screening in asymptomatic children and adolescents with the aim to diagnose PH. The issue at hand seems to lie in the interpretation and reliability of data from interventional studies in children and adolescents with AH and especially in those with PH. There is no doubt that early diagnosis and treatment of AH will prevent the development of CVD and its complications. Moreover, early diagnosis of elevated BP may lead to the introduction of lifestyle changes and subsequent reduction of cardiovascular risk. However, the USPSTF report emphasizes that there is still an insufficient amount of data from prospective and controlled studies in hypertensive children evaluating the effects of antihypertensive treatment. For both ethical and practical reasons a sufficiently long, prospective, placebo controlled trial to evaluate the effects of antihypertensive treatment on cardiovascular morbidity and mortality in hypertensive children, both with secondary AH or with PH, is unlikely. The UPSTF report emphasizes that their statement concerns only PH and that the authors recognize that secondary AH is symptomatic. However, many children with secondary AH are asymptomatic and only BP screening may lead to early diagnosis and treatment.

Nevertheless, the USPSTF statement is a challenge for the paediatric community to report the effects of antihypertensive treatment and to perform prospective studies evaluating the effects of antihypertensive treatments on surrogate markers of CVD and EVA. Moreover, it is a stimulus to define indications for antihypertensive treatment in children with PH based on analysis of cardiovascular risk, including the assessment of TOD and markers of EVA. Two such markers, already widely used in assessment of cardiovascular risk in hypertensive adults, are central BP and central pulse pressure [18, 74].
